# End-of-life tire management: a critical review

**DOI:** 10.1007/s11356-021-16263-6

**Published:** 2021-10-15

**Authors:** Svetlana Dabic-Miletic, Vladimir Simic, Selman Karagoz

**Affiliations:** 1 Faculty of Transport and Traffic Engineering, University of Belgrade, Vojvode Stepe 305, Belgrade, 11010 Serbia; 2 Nottingham Trent University, Nottingham Business School, 50 Shakespeare St, Nottingham, NG1 4FQ UK

**Keywords:** Critical review, End-of-life tires, Waste management, Content analysis, Regulations review, Treatment review, Engineering applications, Network design

## Abstract

Environmental and social awareness are the key elements of the sustainable tire industry. End-of-life tire (ELT) waste flow is an important environmental problem worldwide since it produces severe air, water, and soil pollution issues. Significant advancements have been made in ELT management in the last few years. As a result, ELTs should not only be regarded as waste but also as a source of environmentally friendly materials. Besides, sound ELT management has vital importance for circular economy and sustainable development. Over the last decade, ELT management has attracted many researchers and practitioners. Unfortunately, a comprehensive review of the ELT management area is still missing. This study presents the first critical review of the whole ELT management area. It aims to present an extensive content analysis overview of state-of-the-art research, provide its critical analysis, highlight major gaps, and propose the most significant research directions. A total of 151 peer-reviewed studies published in the journals between 2010–2020 are collected, analyzed, categorized, and critically reviewed. This review study redounds comprehensive insights, a valuable source of references, and major opportunities for researchers and practitioners interested in not only ELT material flow but also the whole waste management area.

**Graphical abstract:**

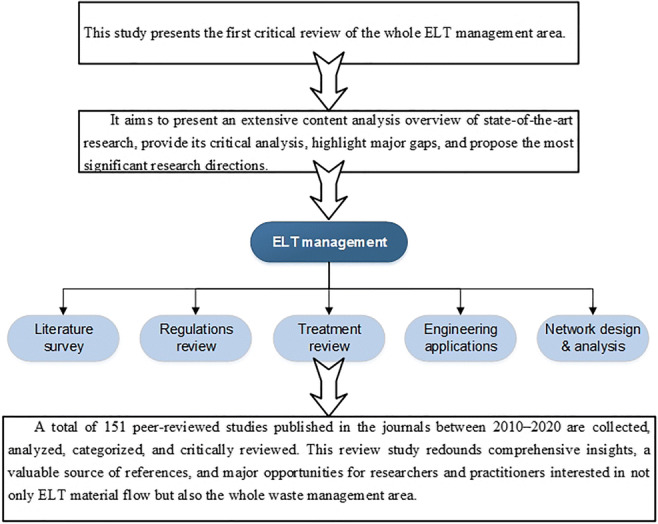

## Introduction

Environmental and social awareness are becoming the key element of the sustainable tire industry. Vehicle tires are a source of pollution throughout their life cycle. End-of-life tires (ELTs^1^) are considered to be one of the most abundant as well as the most attractive waste from an economic point of view. Nearly one billion ELTs are generated worldwide annually and this waste flow is growing dynamically (Wang et al. 2019; Eurostat 2021). ELT waste flow constitutes more than 2% of the total amount of solid waste (Karaagaç et al. 2017). Only in the European market, more than 300 million passenger and truck tires are replaced annually (ETRMA 2019).

ELT waste flow is an important environmental problem worldwide since it produces severe air, water, and soil pollution issues. This waste flow is not biodegradable and belongs to the category of non-hazardous waste. Unfortunately, improper management of ELTs is still a common phenomenon in many economies in transition and developing economies. In fact, nearly one-half of ELT waste flow is disposed of in landfills without any treatment (Junqing et al. 2020). Landfilling of whole and shredded tires might be the most economically sound management option, but it should not be allowed since it presents a major threat to the environment and public health. Since 1999, ELT landfilling has been legally prohibited by the European landfill directive 1999/31/EC (EU. 1999). After that, in 2000, many policies were presented (e.g., 2000/76/EC, 2000/53/EC) which set out more detailed guidelines for ELT management (EU. 2000a, b). In the meantime, there were synchronizations with global regulations. The Waste Framework Directive (WFD) 2008/98/EC provided concepts and definitions related to ELT management (EU, 2008). According to WFD, management options are prevention, minimization, reuse, recycling, energy recovery, and disposal (Son et al. 2011). WFD was a significant step forward in all relevant aspects of waste management, including ELT as a group of end-of-life vehicle (ELV) parts. Based on these regulations, many countries around the world are in the process of developing their regulations to prolong the service life of tires and reduce their negative impact on the environment.

Sound ELT management has vital importance for circular economy and sustainable development. It requires an ecologically efficient and economically effective waste management scheme. ELT management depends on numerous entities (e.g., tire end-users, private and public companies, treatment facilities) and state-of-the-art technologies to convert waste into valuable products. Today, ELT management is a progressive and well-positioned research area. Besides, according to the reports published by the largest associations of tire producers and recyclers (e.g., ETRMA 2019; CRIA - China Rubber Industry Association 2020; JATMA 2020, 2021), significant advancements have been made in sustainable ELT management in the last few years. As a result, ELTs should not only be regarded as waste but also as a source of environmentally friendly materials.

ELT management has attracted many researchers and practitioners. However, to the best of our knowledge, there is no comprehensive review of the whole ELT management area. This study introduces the first critical review of the economic, environmental, and social issues of ELT management. It aims to present an extensive content analysis overview of state-of-the-art research published in the period 2010–2020, provide their critical analysis, highlight major gaps, and propose the most significant research directions. This critical review offers comprehensive insights, a valuable source of references, and major opportunities for researchers and practitioners interested in not only ELT material flow but also the whole waste management area.

The remaining part of the paper is organized as follows: Section 2 describes a review methodology. The results of the literature review are provided in Section 3. The discussion is given in Section 4. The last section presents the conclusions, major gaps, and significant research directions.

## Review methodology

The content analysis is utilized to review the relevant literature. Only peer-reviewed journal papers are reviewed. The search engines, such as Web of Science, Scopus, Taylor and Francis Online, SpringerLink, Wiley Online Library, and Google Scholar, are used to explore the literature.

**Fig. 1:**
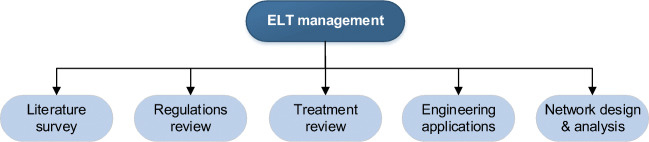
The classification of ELT management studies

The relevant studies are classified into five categories as follows (Fig. 1):

1. Literature survey: Relevant state-of-art reviews are provided in this category. They are evaluated by taking into account their primary scope, coverage of the major categories of ELT management, and the number of reviewed papers.
2. Regulations review: As regulations play an essential role in ELT management, the regulations review studies are overviewed by considering their primary scope and focus, management regulation system, and subject area.
3. Treatment review: Publications that deal with treatment processes and material analysis are analyzed based on their primary scope, treatment type, and ELT application(s).
4. Engineering applications: Researches that explore or provide new applications/markets for the ELT industry from this category. They are surveyed based on their primary scope, considered treatment type, and ELT application(s).
5. Network design and analysis: Studies that are suggesting new methodologies and decision-making approaches for ELT management are grouped into this category. They are reviewed based on their primary scope and focus, considered aspects, and applied method(s).

This classification aims to categorize the relevant studies and to make them more visible for researchers.

## Results

The classification of 151 collected and analyzed researches generates the main framework of the review. Distribution is performed according to the focus of the problems analyzed in the research, i.e., by the field of ELT management to which these papers belong.

### Literature survey

ELT management is becoming more and more interesting and challenging for researchers. Table 1 overviews relevant literature reviews regarding their scope.

**Table 1:** The summary of the literature survey category

**Author(s) and year**	**Scope**	**Category**				**Reviewed papers**
		**RL**	**TR**	**EA**	**NDA**	
Presti (2013)	ELTs in civil engineering	–	–	✓	–	80
Shu and Huang (2014)		–	–	✓	–	~80
Thomas and Gupta (2016)		–	–	✓	–	50
Wang et al. (2018)		–	–	✓	–	~80
Li et al. (2019)		–	–	✓	–	90
Siddika et al. (2019)		–	✓	✓	–	~150
Yadav and Tiwari (2019)		–	–	✓	–	~70
Du et al. (2020)		–	–	✓	–	134
Milad et al. (2020)		–	–	✓	–	110
Picado-Santos et al. (2020)		–	–	✓	–	118
Roychand et al. (2020)		–	–	✓	–	~130
Mokhtar et al. (2012)	Material properties	–	✓	–	–	~60
Williams (2013)		–	✓	✓	–	~80
Danon et al. (2015)	Cost-effective thermochemical process	–	–	✓	–	86
Kumaravel et al. (2016)		–	–	✓	–	~50
Czajczynska et al. (2017)		–	–	✓	–	70
Martinez et al. (2019)		–	–	✓	–	~60
Junqing et al. (2020)		–	✓	✓	–	~120
Santos et al. (2020)		–	✓	✓	–	~100
Quek and Balasubramanian (2012)	Fuel for gas turbines	–	–	–	✓	65
Oboirien and North (2017)		–	✓	–	✓	~55
Sienkiewicz et al. (2017)	ELT management legislative	✓	–	–	–	~100
Uriarte-Miranda et al. (2018)		✓	✓	–	–	62
Sienkiewicz et al. (2012)	Alternative fuels	–	–	✓	–	~70
Ramos et al. (2013)	Treatment comparison	–	✓	–	–	~50
Bharat and Dipak (2014)	LCA	–	✓	–	–	44
Saleh and Gupta (2014)	Tire derived carbons	–	–	✓	–	115
Rowhani and Rainey (2016)	Reuse and energy recovery methods	–	✓	✓	–	~150
Labaki and Jeguirim (2017)		–	✓	✓	–	158
Iraola-Arreguia et al. (2019)	Demineralization	–	✓	–	–	~200
Mmereki et al. (2019)	Innovative treatment methods	✓	✓	–	–	58
Bockstal et al. (2019)	Recycling improvement	–	✓	–	–	~120
Lewandowski et al. (2019)	Reactor efficiency	–	✓	✓	–	~150
***Our review***	*Whole ELT management area*	✓	✓	✓	✓	*151*

ELTs have very wide applications in civil engineering. Presti (2013) investigated the performances of pavements comprising bitumen from ELT recycling. Shu and Huang (2014) outlined the most frequent applications of recycled ELTs as asphalt paving mixtures and lightweight fillers. Rubberized concrete has good mechanical properties and is often an environmentally friendly material used in the construction industry (Thomas and Gupta 2016; Li et al. 2019; Siddika et al. 2019; Roychand et al. 2020). Wang et al. (2018) discussed the eco-efficiency when rubber is used in asphalt mixtures. Yadav and Tiwari (2019) provided an overview of ELT applications in construction for highway and railway embankments, the base material for roads, and as filling material behind a retaining wall. Milad et al. (2020) and Picado-Santos et al. (2020) reviewed ELT applications in asphalt mixtures. Du et al. (2020) investigated the low-temperature performance of asphalt mixtures.

A significant number of review papers is related to the field of pyrolysis as an increasingly common type of ELT treatment. Mokhtar et al. (2012) analyzed microwave pyrolysis for the conversion of materials to energy. In the same year, Quek and Balasubramanian (2012) and Oboirien and North (2017) pointed out the importance of the pyrolitic process for obtaining the gases used in gas turbines. Williams (2013) concluded that tire pyrolysis oil (TPO) is environmentally and economically advantageous to use in comparison to diesel. Martinez et al. (2013) found that pyrolysis represents an attractive thermochemical process, in the meantime. Thereafter, Danon et al. (2015) discussed oils obtained by ELT pyrolysis as chemical resources. Kumaravel et al. (2016) analyzed TPO as an alternative fuel for diesel engines. Similarly, Czajczynska et al. (2017) discussed the characteristics of TPO as a valuable energy source. In 2020, Junqing et al. and Santos et al. provided several studies. Junqing et al. (2020) analyzed carbon black from ELT pyrolysis. Santos et al. (2020) surveyed the feasibility to convert ELTs into chemical components as an alternative recycling method by focusing on TPO.

The European landfill directive 1999/31/EC has been the key driver for improving ELT management. Sienkiewicz et al. (2017) concluded that many countries established corresponding management systems or standards for increasing resource efficiency and reducing negative environmental impacts. As another legislation-based study from a different angle, Uriarte-Miranda et al. (2018) provided the legislative basis for empirical research regarding the assessment of reverse logistics (RL) processes in ELT management.

In today’s environmental conditions, ELTs should be treated not only as a pollutant but also as a valuable raw material (Sienkiewicz et al. 2012). Ramos et al. (2013) analyzed the efficiency, environmental friendliness, and economic viability of technologies for ELT treatment. Regarding ELTs as a group of ELV parts, Bharat and Dipak (2014) provided a review of applications of the life cycle analysis (LCA) methodology. Saleh and Gupta (2014) analyzed the cost-effectiveness of the utilization of ELTs for wastewater treatment. Later on, Rowhani and Rainey (2016) highlighted the importance of ELT recycling and reusing to sustainable environmental stewardship. Labaki and Jeguirim (2017) pointed out thermochemical processes as attractive and practicable ways for recovering energy and materials from ELTs. In 2019, several studies in the same scope were published. Iraola-Arreguia et al. (2019) found the demineralization was one kind of ELT pyrolysis for improving bio-oil quality. Mmereki et al. (2019) discussed an effective ELT management system from technical, environmental, economic, legal, and institutional aspects. Bockstal et al. (2019) analyzed physical and chemical processes for ELT recycling. Lewandowski et al. (2019) described various types of reactors for ELT pyrolysis.

Table 1 shows that the available review papers are focused only on one or two categories of ELT management. Most of the review papers are related to pyrolysis as chemical treatment and ELT applications in civil engineering. Besides, only a few review papers analyzed regulations and network design. Finally, to the best of our knowledge, there is no comprehensive review of the whole ELT management area.

### Regulations review

In terms of worldwide recycling rates of ELTs, the EU takes first place in the list (Gigli et al. 2019). The EU already has appropriate regulations and organizations for ELT management. Besides, there are three different ELT management regulation systems worldwide:

1. Extended producer responsibility (EPR)—tire manufacturers and importers are responsible for ELT collection and treatment
2. Tax system (TS)—ELT collection and treatment are financed through consumer taxes
3. Free-market system (FMS)—local regulations define ELT collection and treatment

In Europe, EPR is the most common in ELT collection and treatment. Santini et al. (2011) found that ELT removal from vehicles needed to fulfill rigorous eco-efficiency targets of the Directive 2000/53/EC. Sohaney et al. (2012) provided analyses of ELT noise in the case of heavy trucks. Bravo and Brito (2012) considered that 5%, 10%, and 15% of the volume of natural aggregate can be replaced by aggregate derived from ELTs. Afterward, Uruburu et al. (2013) highlighted the strong role of non-profit organizations in ELT management. Elnour and Laz (2014) found that lawfully labeling tires could reduce ELT quantity. Hiratsuka et al. (2014) concluded that the Japan automobile tire manufacturer association gave voluntary contributions for the collection of ELTs. In addition to this, Niza et al. (2014) investigated the implementation of the EPR concept in Portugal. Zhang et al. (2016) analyzed legislative barriers and incentive measures to support local enterprises in pyrolysis initiatives. Rodrigues et al. (2016) presented an extended waste input-output model to assess the economic, environmental, and social impacts of the EPR system. Karaagaç et al. (2017) analyzed the degree of ELT recycling in Turkey. Park et al. (2018) concluded that the Colombian EPR system increased the number of ELTs collected over the last 5 years. Malyshkov et al. (2019) analyzed recycling standards for ELTs in Russia. Winternitz et al. (2019) found that the best recycling results were achieved with quantitative targets and clearly defined status of ELT management. Zorpas (2020) promoted the WFD strategies for improving the quality of living conditions, especially in urban areas.

Although EPR is dominant, some studies analyzed TS and FMS regulation systems. Samolada and Zabaniotou (2012) concluded that Greece had nevertheless adopted the EPR system despite the numerous benefits of TS. As a different approach, Sienkiewicz et al. (2012) described different organizational approaches for ELT management in the EU and some possible usages of ELTs as a source of raw materials or alternative fuels. Antoniou and Zabaniotou (2013) outlined general guidelines for EU member states related to ELT disposal. Zabaniotou et al. (2014) continued research to improve pyrolysis due to deficient market analysis, legislative barriers, economic instability, and public acceptance. Later on, Torreta et al. (2015) analyzed treatment and disposal schemes with ELTs in Italy and Romania. Alwaeli (2016) pointed out that Poland was the first European country to introduce an ELT management system, which was initiated by tire manufacturers and importers. Clar-Garcia et al. (2016) studied the European regulations devoted to the reduction of tire noise depending on the age structure of ELTs. Xie et al. (2016) concluded that tire manufacturers took measures to modify the structure of tires to avoid uneven wear. Godlewska (2017) analyzed increased imports of ELTs into Poland. Sienkiewicz et al. (2017) concluded that the establishment of the restrictive regulations, monitoring of improper warehousing, EPR, and TS had a pivotal role in the progress of ELT recovery. In 2018, Uriarte-Miranda et al. (2018) suggested an integrated model by considering regulations and policies in several countries and regions.

A comprehensive summary of the regulations’ review category is presented in Table 2. The table shows that most of the papers are related only to the treatment of ELTs. Besides, only a few papers took into account the strong connection between RL and ELT regulation. Finally, the papers are only focused on the economic and environmental components of sustainability, while the social component is completely neglected.

**Table 2:** The summary of the regulations review category

**Author(s) and year**	**Scope**	**System**			**Focus**			**Subject area**
		**EPR**	**TS**	**FMS**	**COL**	**TRE**	**APP**	
Santini et al. (2011)	ELT recycling and recovery	✓	–	–	–	✓	–	Italy
Sohaney et al. (2012)	Noise from heavy trucks	✓	–	–	✓	✓	–	Europe
Bravo and Brito (2012)	ELT cement granulate	✓	–	–	–	✓	✓	Portugal
Uruburu et al. (2013)	ELT management	✓	–	–	✓	✓	✓	Spain
Elnour and Laz (2014)	ELT quantity reduction	✓	–	–	✓	✓	–	Saudi Arabia
Hiratsuka et al. (2014)	Recycling improvement	✓	–	–	–	✓	–	Japan
Niza et al. (2014)	ELT management	✓	–	–	–	✓	–	Portugal
Zhang et al. (2016)	ELT pyrolysis	✓	–	–	–	✓	–	Global
Rodrigues et al. (2016)	EPR system financing	✓	–	–	–	✓	–	Global
Karaagaç et al. (2017)	ELT quantity	✓	–	–	–	✓	✓	Turkey
Park et al. (2018)	Cost-effectiveness of EPR	✓	–	–	–	✓	–	Colombia
Malyshkov et al. (2019)	EPR and recycling standards	✓	–	–	✓	✓	–	Russia
Winternitz et al. (2019)	EPR system comparison for ELTs	✓	–	–	–	✓	–	Belgium, Italy, The Netherlands
Zorpas (2020)	Energy managing in TPO	✓	–	–	–	✓	✓	EU
Samolada and Zabaniotou (2012)	ELT pyrolysis	✓	✓	–	–	✓	✓	Greece
Sienkiewicz et al. (2012)	Alternative fossil fuels	✓	✓	✓	–	–	✓	EU
Antoniou and Zabaniotou (2013)	Features of ELT pyrolysis	✓	✓	✓	–	✓	✓	EU
Zabaniotou et al. (2014)	Pyrolisis improvement	✓	✓	–	–	✓	–	EU
Torreta et al. (2015)	ELT management schemes	✓	✓	✓	–	✓	–	Italy, Romania
Alwaeli (2016)	ELT recycling level analysis	✓	–	✓	–	✓	–	Poland
Clar-Garcia et al. (2016)	Noise and tire age relationship	✓	✓	–	–	✓	–	EU
Xie et al. (2016)	Tread depth	✓	✓	–	✓	✓	–	Global
Godlewska (2017)	ELT recycling	–	–	✓	–	✓	–	Poland
Sienkiewicz et al. (2017)	Regulations in ELT recovery	✓	✓	–	–	✓	–	EU
Uriarte-Miranda et al. (2018)	RL improvement in ELT management	✓	✓	✓	–	✓	–	Mexico, Russia, Japan, EU

### Treatment review

Inadequate ELT treatment creates major environmental issues. Besides, landfilling might be the most economically sound management option, but it should not be allowed since it presents a major threat to the environment and public health.

ELT treatment and improvement of environmental conditions are some of the most important concerns of sustainable business (Sadiktsis et al. 2012; Ghasemzadeh et al. 2020). In practice, four types of ELT treatment are applied: pyrolysis, recycling, retreading, and energy recovery.

Many researchers have focused on pyrolysis as an attractive ELT treatment. ELT pyrolysis is important for the circular economy since it can provide materials for the production of rubber from which tires are made. Abdul-Raouf et al. (2010) outlined that pyrolysis could be very appropriate for complex materials, such as tires. Sienkiewicz et al. (2012) and Williams (2013) noticed a growing interest in pyrolysis as a technology for producing TPO, char, and gas products. Due to the energy crisis and environmental degradation, Kandasamy and Gokalp (2014) analyzed energy recovery from not biodegradable waste, such as ELTs. Thereafter, Hita et al. (2016) claimed that ELT pyrolysis was the most environmentally friendly option for ELT treatment. Kordoghli et al. (2017) found that the temperature had an important impact on the gas quality. Cherbański et al. (2017) studied the kinetic reaction of ELT pyrolysis. Martinez et al. (2019) examined the performances of carbon black obtained by ELT pyrolysis. Wang et al. (2019) proposed a new protocol for high-value reusing of ELTs. In addition to these, Zhang et al. (2019) analyzed using carbon from ELT pyrolysis for wastewater treatment. Abdallah et al. (2020) found that the produced pyrolysis oil could replace conventional liquid fuels. Buadit et al. (2020) evaluated the potential environmental impacts of an ELT pyrolysis plant in Thailand by using the LCA method. For Sathiskumar and Karthikeyan (2019) and Junqing et al. (2020), pyrolysis is a promising thermochemical process to deal with ELT waste flow.

Recycling is a very common ELT treatment. Recycled ELTs are used in both engineering and non-engineering applications, from raw materials, through semi-finished products to packaging. Gupta et al. (2011a, b) and Derakhshan et al. (2017) pointed out the high potential of recycled ELTs for wastewater treatment applications. Ramarad et al. (2015) analyzed progress in ELT recycling with particular attention to the incorporation of waste tire rubber into polymeric matrices. Kardos and Durham (2015) investigated the properties of rubberized concrete. Depaolini et al. (2017) proved that older ELTs were less favorable due to their chemical composition. In the meantime, Tsai et al. (2017) analyzed ELTs as a supplement of conventional fossil fuel to attain a positive impact on environmental sustainability in Taiwan. Rashid et al. (2019) identified the great potential of using recycled ELTs in concrete as a low- and medium-strength material. Yamashita et al. (2020) analyzed the chemical reactions in the recycling of ELTs.

Retreading is one of the popular approaches for sustainable environmental stewardship of ELTs. This is a process of replacing the spent tread (outer layer of the tire) with a new one by vulcanization to prolong their life cycle exploitation. Retreading is especially beneficial for used truck tires since they could be processed three to four times (Dabić-Ostojić et al. 2014). This type of treatment can have significant environmental and economic sustainability effects (Abdul-Kader and Haque 2011). Bazan et al. (2015) found that retreading offers the most resource-efficient strategy for ELTs because it provided the possibility to save both material and energy. Ortíz-Rodríguez et al. (2017) found the strongest environmental impacts were associated with retreading and recycling of ELTs. Lonca et al. (2018) revealed that extending the lifetime through retreading and recycling improves the circularity of ELTs. Later on, Mrad and El-Samra(2020) analyzed different strategies for ELT management in Lebanon and concluded that retreading is the most economically, environmentally, and socially appropriate treatment.

Energy recovery is an attractive treatment commonly related to the combustion of ELTs in cement kilns. Feraldi et al. (2013) applied LCA to compare different ELT treatment options in the USA context. Aziz et al. (2018) concluded that TPO obtained from pyrolytic reactors could be used in industrial furnaces, power plants, and steam boilers.

The summary of the treatment review category is given in Table 3. As can be seen from this table, ELT pyrolysis and recycling are the most common treatment options. The waste hierarchy emphasizes the reuse and extension of the tire life cycle as a primary ELT management scheme. However, retreading is put into focus in only a few studies. On the other hand, economic efficiency is the primary comparison criterion in most studies. Sustainable ELT treatment should take into account the environmental, economic, and social dimensions of investigated waste flow.

**Table 3:** The summary of the treatment review category

**Author(s) and year**	**Scope**	**Treatments type**				**Application(s)**
		**PYR**	**REC**	**RET**	**ER**	
Abdul-Raouf et al. (2010)	Factors affecting prod. composition	✓	–	–	–	TPO, gas, char
Gupta et al. (2011a, b)	Recycled ELT usage	–	✓	–	–	Wastewater treatment
Feraldi et al. (2013)	Treatment option comparison	–	–	✓	✓	Civil engineering, fuel
Williams (2013)	Pyrolysis product characteristics	✓	–	–	–	TPO, gas, char
Kandasamy and Gokalp (2014)	ELT treatment improvement	✓	–	–	–	TPO, gas
Bazan et al. (2015)	Treatment cost analysis	–	–	✓	✓	Civil engineering
Kardos and Durham (2015)	ELT utilization improvement	–	✓	–	–	Civil engineering
Ramarad et al. (2015)	Polymer blends	–	✓	–	–	–
Hita et al. (2016)	TPO upgrading characteristics	✓	–	–	–	TPO, gas
Cherbański et al. (2017)	ELT pyrolysis kinetics	✓	–	–	–	TPO, rubber
Depaolini et al. (2017)	Recycled rubber characterization	–	✓	–	–	Rubber, playgrounds
Derakhshan et al. (2017)	Recycled ELT usage	–	✓	–	–	Wastewater treatment
Kordoghli et al. (2017)	Product quality	✓	–	–	–	TPO, gas
Ortíz-Rodríguez et al. (2017)	Management option comparison	–	–	✓	✓	Civil engineering, fuel
Tsai et al. (2017)	ELT recycling status	–	✓	–	–	TPO, gas, carbon black
Aziz et al. (2018)	Pyrolitic reactors characteristics	✓	–	–	–	TPO, char
Lonca et al. (2018)	Treatment environmental benefits	–	–	✓	✓	Civil engineering
Zhang et al. (2018)	Pyrolysis efficiency improvement	✓	–	–	–	TPO, carb. black, char
Martinez et al. (2019)	Carbon black production	✓	–	–	–	Carbon black
Rashid et al. (2019)	Rubberized concrete properties	–	✓	–	–	Civil engineering
Sathiskumar and Karthikeyan (2019)	ELT pyrolysis methods	✓	–	–	–	TPO, gas, char
Wang et al. (2019)	High-value temperature pyrolysis	✓	–	–	–	Carbon black, graphene
Zhang et al. (2019)	Pyrolytic carbon preparation	✓	–	–	–	Carbon black
Abdallah et al. (2020)	ELT pyrolysis products analysis	✓	–	–	✓	TPO, gas
Buadit et al. (2020)	Pyrolysis environmental impacts	✓	–	–	–	Energy
Junqing et al. (2020)	Pyrolysis efficiency improvement	✓	–	–	–	TPO, carbon black
Mrad and El-Samra(2020)	Management option comparison	✓	✓	✓	✓	Fuel
Yamashita et al. (2020)	Recycled tire properties	–	✓	–	–	–

### Engineering applications

Improper management of ELTs is still a common phenomenon. It produces serious air, water, and soil pollution issues. Fortunately, there are many environmentally friendly applications of ELT treatment products. Besides, ELT treatment can provide materials that have a wide range of applications from everyday life to commercial and industrial applications. As a result, it is of great interest to explore new applications/markets for the ELT industry.

In 2010, Edinçliler et al. (2010) found that processing techniques and ELT content significantly affect the mechanical properties of used tires-sand mixtures in soils. Fiksel et al. (2011) concluded that the usage of ELTs in civil engineering applications is an environmentally suitable alternative. In the years ahead, Centonze et al. (2012) and Guo et al. (2017) found that there are great possibilities to use steel and rubber from ELTs in civil engineering. Chyan et al. (2013) analyzed ELTs as pollutant removal material from the constructed wetland. Undri et al. (2013), Song et al. (2018), and Ma et al. (2020) analyzed characteristics of limonene as typical valuable chemical products of ELT pyrolysis. Torreta et al. (2015) concluded that ELT treatment has considerable ecological importance. Ayanoglu and Yumrutas (2016) claimed that lime TPO mixture had better results compared to gasoline and diesel fuels for diesel engines. Bičáková and Straka (2016) concluded that some pyrolysis products can serve as heating oil or a source for repairing asphalt surfaces. Fakhri (2016) found that the replacement of the sand by ELT particles in concrete pavement reduced water absorption. Derakhshan et al. (2017) showed the high potential of recycled ELTs for a variety of wastewater treatment applications. Hürdoğan et al. (2017) analyzed how to improve the effects of ELT pyrolysis. Machin et al. (2017) analyzed the energetic valorization of ELTs in Brazil in contexts of job creation, environmental footprint reduction, and electricity generation. Gnanaraj et al. (2018) promoted environmental sustainability through the use of ELTs in the battery industry. After 2018, several studies were published in the same scope. Antoniou and Zorpas (2019) found that ELT waste flow could be a valuable source for energy recovery. Brandsma et al. (2019) found some kinds of paraffin might end up in recycled products. Grioui et al. (2019) analyzed usage of olive oil in ELT pyrolysis for the production of upgraded pyrolytic oil as an alternative fuel. Uyumaz et al. (2019) showed that the TPO-diesel blend gave acceptable performances compared to diesel fuel. Karagoz et al. (2020b) investigated the optimal percentage of TPO in diesel fuel. Liu et al. (2020) provided a brief overview of the engineering properties and environmental effects of recycled ELTs. Narani et al. (2020) concluded that textile fibers from ELTs could enhance the geotechnical characteristics of the expansive soil. Toteva and Stanulov (2020) explored environmentally friendly applications of ELT pyrolysis.

Lately, ELT recycling has attracted more and more attention. Recycled tire rubber is being used in new tires, in tire-derived fuel, in civil engineering applications and products, in molded rubber products, in agricultural uses, recreational and sports applications, and rubber-modified asphalt applications. Thus, the benefits of using rubber-modified asphalts are being more widely experienced and recognized. The incorporation of tires into asphalt is likely to increase, as indicated by an increasing number of researches in this area.

In addition to many well-known, mostly engineering applications, there are many applications where whole, unprocessed ELTs are used. The most interesting and frequent ELT applications are boat protection (Abdul-Kader and Haque 2011), conveyor belts (Aziz et al. 2018), footwear industry (Machin et al. 2017; Aziz et al. 2018; Araujo-Morera et al. 2019), gardening (Fıglali et al. 2015; Singh et al. 2019; Zorpas 2020), lawn grounds (Symeonides et al. 2019), packing material (Thomas and Gupta 2015; Karaagaç et al. 2017; Heidari and Younesi 2020), playground flooring (Bravo and Brito 2012; Girskas and Nagrockienė 2017; Brandsma et al. 2019), thermal and acoustic isolation (Abdul-Kader and Haque 2011; Asaro et al. 2018; Araujo-Morera et al. 2019), vibration reduction on railway tracks (Sol-Sánchez et al. 2014), and wagon wheels (Girskas and Nagrockienė 2017).

The engineering applications category is summarized in Table 4. As can be seen from this table, the most common engineering applications of ELTs are civil engineering and energy sources. ELT recycling is mainly associated with civil engineering applications even though recycled rubber could also be used in other industries. Besides, not enough emphasis is given to explore new fields of applications. Also, there are no researches on ELT applications in logistics activities (e.g., for logistics units in material flows).

**Table 4:** The summary of the engineering applications category

**Author(s) and year**	**Scope**	**Treatment type**		**Application(s)**
		**Pyrolysis**	**Recycling**	
Edinçliler et al. (2010)	Embankment constructions	–	✓	Civil engineering
Fiksel et al. (2011)	Environmental benefits	–	✓	Civil engineering
Centonze et al. (2012)	Modified rubber concrete properties	–	✓	Concrete
Chyan et al. (2013)	Recycled ELT usage	–	✓	Wastewater treatm.
Undri et al. (2013)	Limonene production improvement	✓	–	Limonene
Torreta et al. (2015)	ELT management	–	✓	Civil engineering
Ayanoglu and Yumrutas (2016)	Sulfur amount, environmental protection	✓	–	TPO, fuel
Bičáková and Straka (2016)	Preparation process activities	✓	–	TPO, asphalt
Fakhri (2016)	Modified rubber concrete properties	–	✓	Concrete
Derakhshan et al. (2017)	Recycled ELT usage	–	✓	Wastewater treatm.
Guo et al. (2017)	Modified rubber concrete properties	–	✓	Concrete
Hürdoğan et al. (2017)	ELT pyrolysis effects	–	✓	Fuel
Machin et al. (2017)	Energetic valorization	✓	–	Civil engineering
Gnanaraj et al. (2018)	Anode in lithium-ion batteries	–	✓	Carbon black
Song et al. (2018)	Limonene production improvement	✓	–	Limonene
Antoniou and Zorpas (2019)	TPO in diesel fuel	✓	–	TPO, fuel
Brandsma et al. (2019)	Product characteristics	–	✓	Paraffines
Grioui et al. (2019)	ELT pyrolysis products	✓	–	TPO
Ma et al. (2020)	Limonene production improvement	✓	–	Limonene
Uyumaz et al. (2019)	Sulfur amount, environmental protection	✓	–	TPO, fuel
Karagoz et al. (2020b)	TPO in diesel fuel	✓	✓	TPO, fuel
Liu et al. (2020)	Recycled ELT applications	–	✓	Civil engineering
Narani et al. (2020)	Expansive soil characteristics	–	✓	Civil engineering
Toteva and Stanulov (2020)	Environmentally friendly applications	✓	✓	Energy source

### Network design and analysis

There are a significant number of studies that applied existing models or provided new methodologies to solve problems related to ELT management. In 2010, Sasikumar et al. (2010) developed the mixed-integer non-linear programming (MINLP) model for maximizing the profit of a multi-echelon reverse logistics network for retreading truck tires. Thereafter, Abdul-Kader and Haque (2011) presented an agent-basedmodeling and simulation approach for improving ELT retreading. Aranda et al. (2012) investigated environmentally friendly locations of ELT concept treatment plants.

De Souza and D’Agosto (2013) proposed a value chain analysis for RL supply chain management and explored the financial benefits of sending ELTs to the cement industry. Kannan et al. (2014) presented a decision-making framework to assess ELT management drivers in the Indian context. Dabić-Ostojić et al. (2014) introduced a model based on Bayesian belief networks for making tire retreading-related decisions. Dhouib (2014) used the fuzzy MACBETH to rank remanufacturing alternatives for ELTs.

In 2015, Bazan et al. (2015) presented an economic order quantity-based model for minimizing the costs of the tire retreading industry in Canada, which captured the costs for greenhouse-gasemissions and energy usage. Similarly, Radhi and Zhang (2015) developed MINLP models to simultaneously determine the optimal configuration of ELT remanufacturing networks and return quality decisions under uncertainty. Subulan et al. (2015) formulated a sustainable logistics network design model for tire closed-loop supply chains (CLSC).

Demirel et al. (2016) proposed a mixed-integer linear programming (MILP) model with different scenarios for the number of ELVs in the future. Pedram et al. (2016) presented the MILP model of a multi-echelon CLSC for the tire industry in Tehran, Iran. They used a simple scenario-based approach to represent uncertainties in demand, return rate, and quality of ELTs.

In 2017, Afrinaldi et al. (2017) used a multi-objective genetic algorithm (GA) to determine preventive replacement schedules for bus tires. Amin et al. (2017) formulated a single-objective MILP model for ELT remanufacturing for a real network in Toronto, Canada. Costa-Salas et al. (2017) analyzed the ELT recycling process according to waste collection, processing, and customer zones from a Colombian city. Simic and Dabic-Ostojic(2017) developed an interval-parameter chance-constrained programming model for optimizing long-term purchasing, retreading, and inventory planning in used tire management systems under multiple uncertainties. Simić et al. (2017) introduced an interval-parameter semi-infinitive programming model for used tire management and planning that can successfully handle real-life complex uncertainties.

Several studies dealt with network design and analysis issues of ELT management in 2018. Banguera et al. (2018) provided a MILP model for a reverse logistics network for used tires to meet the EPR national law in Chile. Ebrahimi (2018) developed a stochastic multi-objective programming model for the CLSC network design problem and took into consideration sustainability aspects and quantity discounts under uncertainty. Fathollahi-Fard et al. (2018) formulated a tri-level programming model based on the static Stackelberg game between manufacturers, distributors, and collectors to optimize location-allocation decisions in a tire CLSC. Hajiaghaei-Keshteli et al. (2018) modeled a CLSC of the tire industry as a two-stage stochastic program. Pereira et al. (2018) introduced a forecasting model to estimate the volume and time of used tire returns. Sahebjamnia et al. (2018) formulated a multi-objective MILP model to solve the tire CLSC problem by considering economic, environmental, and social dimensions. Saxena et al. (2018) developed a fuzzy multi-objective mixed-integer programming model to determine organizational and policy insights for a tire remanufacturing SC.

In 2019, Oyola-Cervantes and Amaya-Mier(2019) used the MILP approach to design an RL network for off-the-road tires discarded from the mining industry. Symeonides et al. (2019) compared existing ELT waste management strategies for Cyprus. Zang et al. (2019) conducted an economic analysis of ELT gasification by simulation processes in two types of gasification models: semi-empirical and one-dimensional kinetic models.

In the years ahead, Abdolazimi et al. (2020) proposed a three-objective MILP model for the selection of ELT suppliers for recycling plants based on the time delivery, total profit, and environmental impact. Ghasemzadeh et al. (2020) established MILP formulations for real-life CLSC applications in the Iranian tire industry. Nowakowski and Król (2020) assessed possible scenarios of ELT collection and transportation in Poland including their processing like cutting, baling, and packing. Yu et al. (2020) used the AHP method for investigating characteristics of rubberized asphalt mixtures.

The review of the network design and analysis category is summarized in Table 5. The surveyed ELT management methodologies are rarely tailored for ELTs (Yadollahinia et al. 2018; Xiao et al. 2019; Karagoz et al. 2020a). Only one study took into account the third pillar of sustainable development. The others completely ignored the social aspect of ELT management. Also, the vast majority of the reviewed NDA studies neglected the multi-layer hierarchical nature of ELT management-related problems. Besides, only a few studies integrated legislation, treatments, and applications as three key elements of ELT management. Finally, the available methodologies and decision-making approaches are mostly related only to ELT treatment.

**Table 5:** The summary of the network design and analysis category

**Author(s) and year**	**Scope**	**Goal function(s)**			**Method(s)**	**Focus**		
		**Aspect**	**Type**					
			**Max**	**Min**		**LEG**	**APP**	**TRE**
Sasikumar et al. (2010)	Retreading facility locations	Profit	✓	–	MINLP	–	–	✓
Abdul-Kader and Haque (2011)	Remanufacturing and retreading	Profit, environmental	✓	✓	ABMS	✓	–	✓
Aranda et al. (2012)	ELT treatment plant locations	Distance	–	✓	CWA	✓	–	✓
De Souza and D’Agosto (2013)	RL for ELT	Cost	–	✓	VCA	–	✓	–
Kannan et al. (2014)	ELT management drivers	–	–	–	ISM	✓	–	–
Dabić-Ostojić et al. (2014)	Tire retreading	Distance	–	✓	BN	✓	✓	✓
Dhouib (2014)	ELT remanufacturing strategy	–	–	–	Fuzzy MACBETH	–	✓	✓
Bazan et al. (2015)	Tire RL inventory management	Cost	–	✓	EOQ	–	✓	✓
Radhi and Zhang (2015)	Remanufacturing production network	Profit	✓	–	MINLP	✓	✓	✓
Subulan et al. (2015)	Tire manufacturing CLSC	Profit, environmental	✓	✓	MILP, IFGP	–	–	✓
Demirel et al. (2016)	ELV reverse logistics network	Cost	–	✓	MILP	✓	–	✓
Pedram et al. (2016)	Tire management CLSC	Profit	✓	–	MILP	–	–	✓
Afrinaldi et al. (2017)	Tire replacement schedule	Profit, environmental	✓	✓	MOP, GA	–	–	✓
Amin et al. (2017)	Tire management CLSC	Profit	✓	–	MILP, DT	–	–	✓
Costa-Salas et al. (2017)	Tire recycling network	Profit, environmental	✓	✓	DES, MOP	–	–	✓
Simic and Dabic-Ostojic(2017)	Used tire management and planning	Profit	✓	–	IPCCP	–	–	✓
Simić et al. (2017)	Used tire management and planning	Profit	✓	–	IPSIP	–	✓	✓
Banguera et al. (2018)	RL for used tires	Cost	–	✓	MILP	✓	–	✓
Ebrahimi (2018)	Tire management CLSC	Cost, environmental, awareness	✓	✓	SMOP, ε-constrain	✓	–	✓
Fathollahi-Fard et al. (2018)	Tire management CLSC	Cost	–	✓	TLP, SA, VNS, KA, SFS	–	–	✓
Hajiaghaei-Keshteli et al. (2018)	Tire management CLSC	Cost, risk	–	✓	TSSP, PSO, GA	–	✓	✓
Pereira et al. (2018)	ELT quantity forecasting	–	–	–	TFM, ARIMA	–	–	✓
Sahebjamnia et al. (2018)	Tire management CLSC	Cost, environmental, social	–	✓	MOP, MILP, GA, SA, TAS, RDA, WWO	–	–	✓
Saxena et al. (2018)	Tire remanufacturing SC	Profit, coverage, flexibility, env.	✓	✓	FMOMIP	–	–	✓
Oyola-Cervantes and Amaya-Mier(2019)	Off-the-road tire RL	Profit	✓	–	MILP	–	✓	✓
Symeonides et al. (2019)	ELT strategy selection	–	–	–	SWOT	–	–	✓
Zang et al. (2019)	ELT gasification	Cost	–	✓	TCM	–	✓	✓
Abdolazimi et al. (2020)	Tire management CLSC	Delivery, profit, environmental	✓	✓	MOP, MILP, ε-constrain	–	–	✓
Ghasemzadeh et al. (2020)	Tire management CLSC	Profit, environmental	✓	✓	MILP, ε-constrain	–	✓	✓
Nowakowski and Król (2020)	ELT collection	–	–	–	AHP, PROMETHEE	✓	–	✓
Yu et al. (2020)	CRMA mixing sequence	–	–	–	AHP	–	✓	

## Discussion

**Fig. 2:**
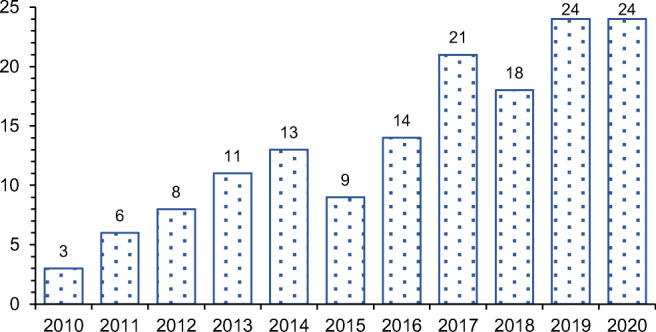
The distribution of papers per year across the period 2010–2020

The annual distribution of researches in the ELT management area between 2010 and 2020 is given in Fig. 2. As can be seen from Fig. 2, there is a significant increase in the number of researches from 2017. Besides, over the last 2 years, almost one-third of the analyzed papers have been published. This indicates a growing interest in the ELT management area.

**Fig. 3:**
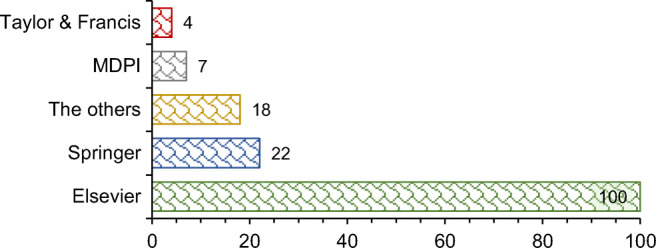
The distribution of the reviewed studies based on the publisher

This presented critical review comprehensively analyzes state-of-the-art studies published by the world’s largest publishers. Figure 3 presents the distribution of the reviewed studies based on the publisher. The primary publisher for the ELT management research area is Elsevier with 100 publications and a 66.2% share. Twenty-two studies were published by Springer (14.6% share), seven studies were published by MDPI (4.6% share), and four studies were published by Taylor & Francis (2.6% share). The other 18 studies, which is less than 12% of the analyzed papers, were published by some other publishers such as ASME and SAGE.

**Fig. 4:**
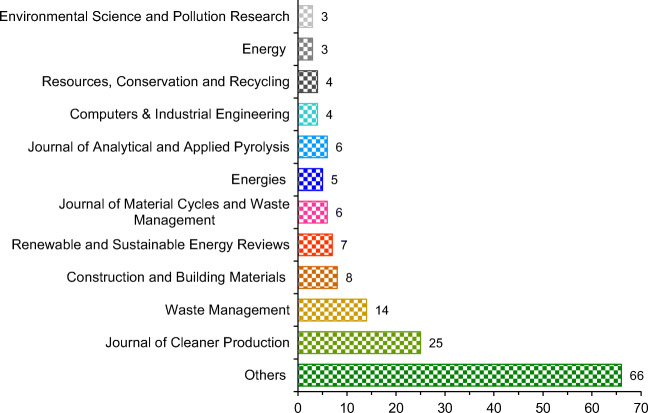
The distribution of the reviewed studies based on the source of publication

This research presents a comprehensive overview of 151 papers published in 69 peer-reviewed journals. Figure 4 depicts the distribution of the reviewed papers based on the source of publication. The largest number of papers were published in the Journal of Cleaner Production (25 publications) and Waste Management (14 publications), i.e., 25.8% of all collected papers. A significant number of contributions were also published in journals such as Construction and Building Materials (8 publications), Renewable and Sustainable Energy Reviews (7 publications), and Journal of Material Cycles and Waste Management (6 publications). The remaining 66 papers were published in 58 different journals.

**Fig. 5:**
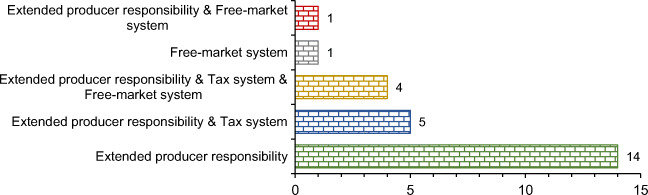
The ELT management regulation system of the regulations review category

The EU and Japan have the most developed regulations in the field of ELT management. However, only three papers investigated ELT management regulation systems on a global level (Table 2). Figure 5 presents the number and percentage of the regulations review papers based on the ELT management regulation system. EPR system is favored in almost all papers, as the most common regulation system for ELT management. A large number of studies (14 out of 24) advocates exclusively this system. This indicates the importance of the fact that tire manufacturers are increasingly taking responsibility when products end their service life. Such practice has positive effects on environmental, economic, and social sustainability.

**Fig. 6:**
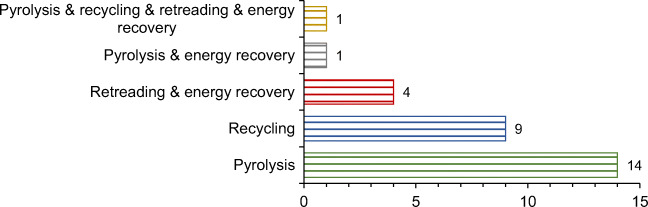
The distribution of the treatment review category by ELT treatment

Figure 6 gives the distribution of the treatment review papers based on investigated ELT treatment. As can be seen from Fig. 6, pyrolysis is the most common type of ELT treatment since it is investigated in the majority of the treatment review papers. More than 80% (23 out of 29) of papers investigate recycling and/or pyrolysis, although in the waste hierarchy, extending the life of tires is an imperative of sustainable treatment. Besides, retreading is investigated in only five studies even though it is fully in line with the environmental, economic, and social dimensions of ELTs. These facts indicate that there are many challenges for future research in the treatment review category.

**Fig. 7:**
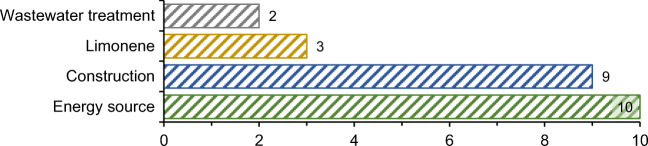
The distribution of the engineering applications category

After appropriate ELT treatment, certain materials are obtained which are later used as a substitute for raw materials. In almost all papers related to ELT management, their applications are analyzed, discussed, and evaluated. Figure 7 provides the distribution of the engineering applications category based on provided ELT waste flow applications. According to Fig. 7, 24 papers deal exclusively with applications, with ELTs being analyzed as an energy source in more than 41% of the engineering applications category, while 37.5% of this category prefers construction-related applications. These results are directly related to investigated ELT treatment, where pyrolysis and recycling are most commonly used.

**Fig. 8:**
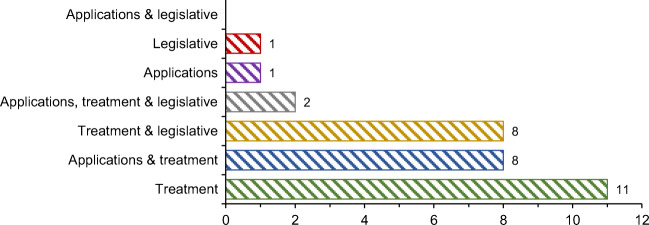
The research focus of the network design and analysis papers

In a significant number of analyzed papers, optimization approaches are applied to improve ELT management. Figure 8 presents the number and percentage of the network design and analysis papers based on their research focus. From Fig. 8, it can be noticed that in 35.5% (11 out of 31) of the network design and analysis papers, the authors developed new methodologies and decision-making approaches that only deal with ELT treatment. In addition to treatment, legislation is also respected in 25.8% (8 out of 31) of papers. In most of the developed new methodologies and decision-making approaches, at least two key elements of ELT management are respected: most often applications and treatment as well as treatment and legislative. However, only two studies integrated applications, treatments, and legislation, as three key elements of ELT management. Based on these indicators, it can be outlined that the vast majority of network design and analysis papers failed to take into account all three key elements of ELT management.

## Conclusions

This study presents the first critical review of the ELT management area. Relevant peer-reviewed publications in the journal in the period 2010–2020 are collected, analyzed, categorized, and critically reviewed. As a result, an extensive content analysis overview of 151 state-of-the-art studies is provided.

There is a significant increase in the number of studies after 2017. Besides, almost one-third of the reviewed papers were published in the last 2 years. These indicators highlight the growing importance of ELT management. On the other hand, the reviewed studies were published in 69 peer-reviewed journals. The major publishers for the ELT management area are Elsevier (66.2% share) and Springer (14.6% share). The primary publication outlets are the Journal of Cleaner Production and Waste Management. The secondary publication outlets are Construction and Building Materials, Renewable and Sustainable Energy Reviews, and Journal of Material Cycles and Waste Management.

Most of the papers are related only to the treatment of ELTs. Pyrolysis and recycling are the most common ELT treatment options, while economic efficiency is the primary comparison criterion. The EU and Japan have the most developed regulations in the field of ELT management. From the regulation aspect, the EPR management system is most often implemented. This indicates that environmental awareness is becoming increasingly important in ELT management.

According to the performed review, the major gaps and significant research directions are as follows:

i. Sustainable waste management should take into account the environmental, economic, and social dimensions of ELT material flow. The social dimension, as the third pillar of sustainable development, is mainly ignored in the available studies. Also, social indicators (i.e., affected population, customer satisfaction, health and safety practices, job opportunities and unemployment, local influence and development, occupational hazards, public awareness level, safety management, etc.) should be taken into account in future research efforts to generate comprehensive guidelines for relevant decision-makers.
ii. Regulations play an essential role in ELT management. However, they have not been sufficiently respected in a significant portion of the previous studies. This negative trend is especially visible in the available network design and analysis research efforts. Future studies on new methodologies and decision-making approaches for ELT management should cover all three key elements of ELT management, i.e., legislation, treatments, and applications. Besides, future models need to be specifically tailored for ELTs.
iii. Little has been done to investigate ELT management regulation systems on a global level. Also, the influence of EPR, TS, and FMS regulation systems on tire material circularity is missing. Besides, critical enablers and barriers for tire material circularity still have to be revealed.
iv. The waste hierarchy emphasizes the reuse and extension of the tire life cycle as a primary ELT management scheme. Retreading is explored in only a few studies. It is on top of the waste management hierarchy. This insufficiently researched management scheme, which is fully in line with the environmental, economic, and social dimensions of ELTs, needs to be put into focus in future studies.
v. ELT recycling is mainly associated with civil engineering applications even though recycled rubber could also be used in many other areas. New fields of applications of recycled ELTs have to be explored and well-documented to minimize ELT waste flow, e.g., innovative applications for thermal and acoustic isolation, vibration reduction, packaging as well as widespread utilization in the footwear industry and logistics activities.
vi. Many studies in the literature deal with network design and analysis of ELTs; however, there are significant gaps in this scope. Only a few studies propose a strategic approach for the remanufacturing process of ELTs. In addition, very few studies focus on the social and environmental impacts of ELTs. Since uncertainty is one of the crucial factors in an effective network design process, there is no significant number of studies in the literature that takes uncertainty into account. As sustainability and resilience are key factors for the future of a realistic project, researchers can include these factors in future studies related to ELT management. Last but not least, reconcilement of conflicting goals in ELT optimization models could be an interesting topic to deal with for future researchers since economic, social, and environmental objectives tend to trade-off in waste management models.
vii. Short- and long-term effects of external impact factors on the tire industry must be comprehensively assessed. Also, possibilities for increasing supply chain resilience should be extensively explored. Besides, a key challenge is to provide a set of alternative solutions that can serve as a rule of thumb for supply chain managers under medical crises, like the COVID-19(coronavirus) outbreak. To help researchers and practitioners in related future efforts, we introduce the novel concept of a “socially resilient supply chain”, where social resilience is defined as the ability of a sustainable supply chain to timely, eco-efficiently, and cost-effectively recover from social disruption events. It should be outlined that this research avenue deserves special attention.

The presented critical review provides comprehensive insights, a valuable source of references, and major opportunities for researchers and practitioners interested in not only ELT material flow but also the whole waste management area.

## Data Availability

All authors consent when it is published.

## Footnotes

1 All abbreviations are provided in the Appendix.

## Abbreviations

ABMS: Agent-based modeling and simulation
AHP: Analytic hierarchy process
APP: Application
ARIMA: Autoregressive integrated moving average model
BN: Bayesian network
CLSC: Closed-loop supply chain
COL: Collection
CRMA: Crumb rubber modified asphalt
CWA: Clarke-Wright algorithm
DES: Discrete event simulation
DT: Decision tree
EA: Engineering applications
ELT: End-of-life tire
ELV: End-of-life vehicle
EOQ: Economic order quantity
EPR: Extended producer responsibility
ER: Energy recovery
EU: European union
FMOMIP: Fuzzy multi-objective mixed-integer programming
FMS: Free-market system
GA: Genetic algorithm
IFGP: Interactive fuzzy goal programming
IPCCP: Interval-parameter chance-constrained programming
IPSIP: Interval-parameter semi-infinitive programming
ISM: Interpretive structural modeling
KA: Keshtel algorithm
LCA: Life cycle analysis
LS: Literature survey
MACBETH: Measuring attractiveness by a categorical based evaluation technique
MCDM: Multi-criteria decision-making
MILP: Mixed-integer linear programming
MINLP: Mixed-integer non-linear programming
MOP: Multi-objective programming
NDA: Network design and analysis
PROMETHEE: Preference Ranking Organization METHod for Enrichment of Evaluations
PSO: Particle swarm optimization
PYR: Pyrolysis
RDA: Red deer algorithm
REC: Recycling
RET: Retreading
RL: Reverse logistics
RR: Regulations review
SA: Simulated annealing
SFS: Stochastic fractal search
SMOP: Stochastic multi-objective programming
SWOT: Strengths, weaknesses, opportunities, threats
TAS: Tabu search
TCM: Technical cost modeling
TFM: Transfer function model
TLP: Tree-level programming
TPO: Tire pyrolysis oil
TR: Treatment review
TRE: Treatment
TS: Tax system
TSSP: Two-stage stochastic programming
VCA: Value chain analysis
VNS: Variable neighborhood search
WFD: Waste Framework Directive
WWO: Water wave optimization
